# The Dental Laboratory

**Published:** 1895-07

**Authors:** 


ARTICLE III.

### THE DENTAL LABORATORY.

### THE USE OF TOOLS.

Nothing more conclusively shows the difference between a good experienced workman and a novice than the manner in which he uses tools and other appliances, in the dental as well as any other work-room. Not only is this apparent in the handling of the same while doing his work, but it is also in a more marked degree shown by his improper selection of tools for purposes they are not intended for.

The rougher portions of our work will here be referred to more particularly, the writer having found, from experience of student life, that it is in this section that one has to look for the most damage. For example, in drawing wire, instead of using a pair of draw-tongs, a small pair of pliers is taken, a strain is put on the handles greater than they are intended to bear, and as a consequence one of the blades snap off. Again, a small pair of nippers, sufficiently strong for cutting ordinary dental plate, is used to cut something three times as thick, perhaps a tooth on a zinc model. What is the consequence? A fracture. Another instance occurs while drawing wire; it is not dried after annealing, or the draw-plate is put in a damp place, and not properly oiled after use; it thus becomes rusty and inaccurate. Again, while rolling gold, the ingot of metal is not cleaned from flux, or is not dry; as a consequence, a valuable set of rollers is spoilt in a very short time.

Tempered broaches are used as if they would bear any strain and are frequently broken the first time of using.

These are some of the abuses of the use of tools, and all of them could be avoided, if one were only to think for a moment that if great force is to be employed it requires a strong instrument, or tool, in proportion. Accidents will happen sometimes, but with a little care and thought, such as have been named, can be avoided.

The tools and appliances in the laboratory should be kept in a state of efficiency. New saws in frames when required, sculptors sharp, and drills in good condition. All these matters should be attended to directly; work is slack, or there is idle time; by these means, when busy again, one is able to go on with the work with comfort and despatch.

In conclusion, it should be mentioned that all tools, lathes and appliances requiring it should be kept clean, bright and well oiled, either with vaseline or goose-grease; both these being lubricants that do not thicken or get sticky.

To clean lathes, etc., when dirty, they should be rubbed with paraffine oil.

### WORK-ROOM ECONOMY.

To study economy while doing his work is one of the earliest principles that should be instilled into the mind of the young beginner, and he should be taught to so habituate himself to carry out the necessary details that in time it becomes natural to him to do so without an effort.

Foremost among the many channels of waste in the dental work-room is the gold drawer; this should be provided with a hare's-foot or a large badger-hair tooth-brush, that being much softer than the ordinary ones.

When gold is being filed, the drawer should be kept wide open, so that, if a rough file is being used, the small particles of gold do not fly over the edges of the drawer.

Now the drawer cannot be kept properly open if the workman sits too close to his bench-pin; therefore this part of his education should not be neglected. Again, too great stress cannot be laid on the fact that the gold drawer is not the place for tools of any kind.

If files are used, a slight tap on the bench-pin will detach any loose filings that may be adherent to them, and then they should be placed on the bench in front of or at the right side of the work-man.

Should they be put in the gold drawer, particles of gold attach themselves to the handles, after they have been in the warm hand, and if they are not brushed clean each time, before using again, (which in itself is a waste of time), considerable losses of gold will take place.

Drills and broaches should be passed through the brush also, and all work after filing, as well as the bench-pin, should be brushed before placing the case on the model pain.

By attending to these apparently small matters, a considerable saving will take place in the course of a twelve-month. With all one's care it is impossible to avoid some waste, but one can account for a large percentage of it, by carefully saving all the dust that collects on the bench, the dirt off the floor, the old rags that one uses for wiping cases, all the sediment that collects in the water-troughs of the lathes, all the washings of the hands after polishing cases. All these small matters may be overlooked by the inexperienced beginner, and thrown away. The author does not advocate keeping these "sweeps," as they are called, separate, as it entails much extra work, which in the long run does not pay. When a sufficient quantity, say a good-sized barrel full, has been collected, it should be sold to some respectable refiner. To obtain information on this point, it is as well to consult some old dental friend. The author's experience is that refiners are not all alike, in the way they treat their customers. A notable example of this was brought under the writer's notice quite recently. It was a case in which for six or seven years the average for the floor "sweep" did not amount to a pound per annum. On the recommendation of a friend, a new refiner was tried, with the result that a cheque for nearly eight times the former amount was received. Taking into consideration that the average of work was about the same, one must naturally conclude that there must have been something wrong somewhere.

Another important consideration is in the consumption of gas, for one may find it flaring away, not only before, but long after it has served its purpose; this not only represents a money loss, but it also tends to vitiate the surrounding atmosphere, and is detrimental to health.

Again, one might mention the waste that takes place with use of plaster of Paris. This might be avoided if one noticed the amount of plaster necessary for each operation; in a very short time a fairly accurate idea would be gained of the amount required; and much waste would be avoided.

Now we arrive at another important item, and that is, a loss of time.

Speaking of this, we do not wish it to be understood that we would curtail the time necessary to do the work effectively and well, but, on the contrary, would allow the maximum time if it ensured the work being more perfect. But a great amount of time is lost, especially with the young beginner, in not rectifying an error at once. For instance, if a clasp does not please us by its fit or shape, or if the position of a pin, for a tube tooth, does not seem to us quite as it should be, it is quite as well to get into the habit of altering it at once, than by thinking over it and so lose more time than it would take to make the necessary alteration two or three times over.

Then again, the workman should always "be doing." If he is waiting for the plate to clean, or the case to cool, he can be going on with the clasps, backing the teeth, or putting his tools into an efficient condition. This is the great secret of rapid working.

While we would advocate diligence in the work, we would at the same time point out that it is not the noisy workman who gets through the most work. Noise is a thing that should on every occasion be avoided. Much noise may be prevented when any hammering has to be done on the bench, by resting the anvil, or block, upon a bag of sand or a thick layer of rags. Swaging up the plate with the model resting on the bench should be considered a gratuitous annoyance to everyone in the work-room, and is to be condemned, considering how easy it is to avoid it.

*The use of the dental engine in the laboratory:* The importance of this appliance in the work-room cannot be over-estimated, and if used in a careful and intelligent manner, it will last a considerable time without getting out of order.

For fine-fitting tube teeth to a plate, for letting down a crown or pivot tooth on to a root, for such fine operations as fitting porcelain facing to bicuspid crowns, it has no equal; the minute wheels, cones and sticks of carborundum which can be used with it enable one to produce the most perfect results.

Again, for repairing split vulcanite cases, after making a little plaster model of the palate of the case, a fissure bur in the engine can be used to produce a new clean surface to the edges of the fissure, by first of all running the bur through the plate and drawing it from one end of the split to the other, after which lateral cuts can be made on each side for the further strengthening of the new rubber.

For hollowing out spaces in a rubber plate for the introduction of a strengthener, for easing cases over roots; for removing the drugs on rubber cases corresponding to the necks of the teeth, a large rose bur is most suitable and does the work neatly and well.

For removing a tooth or gum block from a case, a small bur can be used to work around the pins and remove the block without damage to the case.

For polishing the inside of gold plates, clasps, and round about the backs of the teeth, also in the indentations of the rugæ, the engine can be employed with effect, as the extremely small appliances used with it can be introduced into the most constricted space.

One is also enabled to mount a small circular saw, which can be used as well as a wheel for removing a metal back from a plate.

These are a few samples of the uses the engine can be put to, and for which it is especially adapted.

To make the little chucks to carry the small wheels, etc., a piece of round polished steel wire is obtained the same thickness as an engine bur. The wire is nicked deeply at suitable lengths with a file, and snapped off, and then the ends are filed smooth.

These pieces are held very firmly in a Hodge hand-piece.

We now take some German silver tube that will just fit the wire. Cut the tube into lengths, of an inch long, and file each end level. We must next slip one of these tubes onto the wire, leaving the end protruding about one-eighth of an inch. We solder the tube to the wire with silver solder. By this means a shoulder is formed for the wheel to rest against.

A porte-polisher for carrying points of corundum, or carborundum, can also be very simply made, by soldering a tube on to the steel wire, letting the end of the tube project about half to three quarters of an inch. This projecting portion can be tapered a little and split. To fix the carborundum point, melt a little shellac into the tube, then make the carborundum point hot, and press it into the melted shellac.

THE MATERIALS USED IN THE DENTAL LABORATORY.

These may be grouped in the following order:

*For moulding in sand;* Sand or loam, pumice, brick-dust, French chalk, lycopodium and common resin.

The latter is used to give a carbon surface to a sand or other cast. A stick of ordinary firewood is taken and either dipped into melted resin, or the resin is melted over a flame and dropped on to the wood. It is then set alight, and holding the sand mould over the smoke of the resin, the surface of the sand is coated with a black deposit in a few moments. By coating the sand or other mould in this manner a better surface is obtained to the resulting model.

*For polishing purposes:* Pumice, rouge, whitening, water-of-Ayr stone, sandpaper, and for polishing steel, emery and oil.

The art of polishing is to produce first of all a perfectly smooth surface, free from scratches and irregularities. This should be accomplished with the sculptor and smooth file. Afterwards sandpaper and water-of-Ayr stone must be used. A good surface should be put on a case before it is taken to the lathe, so that only a moderate amount of rubbing with pumice and oil (if a gold plate), or pumice and water (if a vulcanite plate), need be used, and the final finish may be given with rouge, or whitening and water.

*For modeling purposes:* Beeswax, yellow and white, paraffine, resin, and, as a coloring matter for the wax, carmine.

*The coarse metals in general use for casting purposes are*: Lead, zinc, tin, type metal, fusible metal, meter metal and Sullivan's cement.

Sullivan's cement may be prepared in the following manner: Make a saturated solution of sulphate of copper in boiling water. Then place in the solution several pieces of sheet iron. The copper will be immediately precipitated. Pour off the water and wash the precipitate several times in weak sulphuric acid; this will dissolve out all the zinc. Now add to the copper precipitate a sufficient quantity of mercury to amalgamate with and form it into a stiff paste. The surplus mercury is expressed from the mass by squeezing in a piece of chamois leather. It should now be moulded into small pellets and allowed to harden.

Sullivan's cement comes in for many useful purposes, such as making models for crowns, or unwearable teeth for plaster models. If used for these purposes, it should be softened and pressed into the cavities representing the teeth in the impression. The impression is then filled up with plaster of Paris and allowed at least eight hours to harden, before it is placed in hot water to remove the composition. We shall thus have a plaster model with teeth of Sullivan's cement.

*Materials for models:* Plaster of Paris of the finest quantity should be used for this purpose; this is known as "superfine." For other purposes than models, a lower grade of plaster may be used.

To prepare a plaster model for a gold plate, it should be carefully—that is, slowly—dried, and then, while still warm, placed in hot melted stearine, or in melted wax and resin, or it may be varnished with methylated brown spirit varnish. The object of thus preparing it is to harden the model so that it is less liable to be rubbed during the swaging of the plate and fitting of the clasps, and it also makes the model nicer to handle.

*Materials used as a parting medium between—*

1st. Two surfaces of composition. Vaseline, French chalk or soap.

2d. Between two surfaces of wet plaster. Soap or vaseline, preferably the former.

3d. Between hard rubber and a plaster surface. French chalk, silicate of soda; thick tin foil or meter metal, or, before the rubber is vulcanized, French chalk or a piece of linen. This latter should be dipped in hot water before applying it.

*Acids used for cleaning plates:* For gold, hydrochloric acid; for silver and dental alloy, sulphuric acid.

These may be used slightly diluted, and kept in an ordinary gallipot fitted with a cover. When it is found necessary to boil the case out in sulphuric acid a copper dish may be used for that purpose, and the fumes of the acid allowed to escape up the chimney.

*Materials used as a flux for soldering purposes:* Borax for gold, silver, platinum, dental alloy, copper and brass. Zinc chloride for tin, lead, zinc or pewter.

*Chemicals used for collecting and purifying gold filings:* Potassium carbonate, potassium nitrate, sodium chloride, hydrargyrum bichloride. When the filings are collected into a button, this should be remelted with borax. —*British Journal of Dental Science.*

